# Improved Clinical Workflow for Whole-Body Patlak Parametric Imaging Using Two Short Dynamic Acquisitions

**DOI:** 10.3389/fonc.2022.822708

**Published:** 2022-04-28

**Authors:** Hui Wang, Ying Miao, Wenjing Yu, Gan Zhu, Tao Wu, Xuefeng Zhao, Guangjie Yuan, Biao Li, Huiqin Xu

**Affiliations:** ^1^ Department of Nuclear Medicine, First Affiliated Hospital of Anhui Medical University, Hefei, China; ^2^ Department of Nuclear Medicine, Ruijin Hospital, Shanghai Jiao Tong University School of Medicine, Shanghai, China

**Keywords:** dynamic PET, whole-body parametric imaging, Patlak, ^18^F-FDG, positron emission tomography/computed tomography (PET/CT)

## Abstract

**Objective:**

We sought to explore the feasibility of shorter acquisition times using two short dynamic scans for a multiparametric PET study and the influence of quantitative performance in shortened dynamic PET.

**Methods:**

Twenty-one patients underwent whole-body dynamic ^18^F-FDG PET/CT examinations on a PET/CT (Siemens Biograph Vision) with a total scan time of 75 min using continuous bed motion for Patlak multiparametric imaging. Two sets of Patlak multiparametric images were produced: the standard MR_FDG_ and DV_FDG_ images (MR_FDG_-_std_ and DV_FDG_-_std_) and two short dynamic MR_FDG_ and DV_FDG_ images (MR_FDG_-_tsd_ and DV_FDG_-_tsd_), which were generated by a 0–75 min post injection (p.i.) dynamic PET series and a 0–6 min + 60–75 min p.i. dynamic PET series, respectively. The maximum, mean, and peak values of the standard and two short dynamic multiparametric acquisitions were obtained and compared using Passing–Bablok regression and Bland–Altman analysis.

**Results:**

High correlations were obtained between MR_FDG_-_tsd_ and MR_FDG_-_std_, and between DV_FDG_-_tsd_ and DV_FDG_-_std_ for both normal organs and all lesions (0.962 ≦ Spearman’s rho ≦ 0.982, *p* < 0.0001). The maximum, mean, and peak values of the standard and two short dynamic multiparametric acquisitions were also in agreement. For normal organs, the Bland–Altman plot showed that the mean bias of MR_FDG-_max, MR_FDG-_mean, and MR_FDG-_peak was -0.002 (95% CI: -0.032–0.027), -0.002 (95% CI: -0.026–0.023), and -0.002 (95% CI: -0.026–0.022), respectively. The mean bias of DV_FDG-_max, DV_FDG-_mean, and DV_FDG-_peak was -3.3 (95% CI: -24.8–18.2), -1.4 (95% CI: -12.1–9.2), and -2.3 (95% CI: -15–10.4), respectively. For lesions, the Bland–Altman plot showed that the mean bias of MR_FDG-_max, MR_FDG-_mean, and MR_FDG-_peak was -0.009 (95% CI: -0.056–0.038), -0.004 (95% CI: -0.039–0.031), and -0.004 (95% CI: -0.036–0.028), respectively. The mean bias of DV_FDG-_max, DV_FDG-_mean, and DV_FDG-_peak was -8.4 (95% CI: -42.6–25.9), -4.8 (95% CI: -20.2–10.6), and -4.0 (95% CI: -23.7–15.6), respectively.

**Conclusions:**

This study demonstrates the feasibility of using two short dynamic scans that include the first 0–6 min and 60–75 min scans p.i. for Patlak multiparametric images, which can increase patient throughout for parametric analysis.

## Introduction

Positron emission tomography/computed tomography (PET/CT) is widely used in clinical oncology for diagnosis, staging, and therapy monitoring (1–3). ^18^F-fluorodeoxyglucose (FDG) has been the dominant PET tracer in oncology for over 40 years (4). In current clinical practice, FDG PET/CT imaging is performed at a predefined time point, usually 60 min post FDG injection (5, 6). The acquired data are reconstructed to produce a conventional (static) PET image. Static PET images are quantified by the standardized uptake value (SUV), which is a semi-quantitative measure of glucose uptake (7, 8). However, PET tracer distribution is a dynamic process while the SUV is derived from a static single snapshot of FDG after it is equilibrated between the blood plasma and tissue. Therefore, the SUV does not allow any conclusions regarding the rate of irreversible uptake to observed tracer uptake to be drawn. Static PET imaging captures not only FDG-6-P retained in glucose metabolizing tissue but also the substantial activity of unbound or free FDG in tissue and blood vessels. Thus, the SUV does not provide a reliable measurement of the kinetics of FDG in lesions, and it cannot differentiate malignant tumors from physiological or benign processes such as inflammation (9–11).

The dynamic course of the FDG spatial distribution in the targeted tissues may reveal highly useful clinical information about the tissue’s metabolic properties (12, 13). The streamlined graphical analysis called the Patlak method coupled with a plasma input function to estimate the tracer uptake rate *K*_i_ (slope) and the total blood distribution volume *V* (intercept) was invented in 1983 (14). Patlak modeling has been validated and developed for the generation of parametric images (15, 16). With the rapid development of PET/CT scanners, a fully automated multiparametric whole-body (WB)-PET based on FlowMotion workflow and direct Patlak reconstruction from list-mode data was developed by Siemens. The parameters are the *K*_i_, which is the rate of irreversible uptake, and the Patlak intercept (DV), which is the apparent distribution volume of the non-metabolized FDG. In recent studies, dynamic multiparametric images may achieve equivalent or superior lesion detectability with reduced false-positive rates when complementing SUV imaging (17, 18), which highlighted the potential role in the initial tumor diagnosis and characterization (19, 20). The Patlak model requires tissue time–activity curves (TACs) after equilibrium of FDG between blood plasma and tissue, and knowledge of the input function from the start of injection (21–23). Therefore, a Patlak dynamic scanning protocol typically starts from the radiotracer injection until after FDG equilibrium. Accordingly, the patients need to be injected on the scanner bed and remain motionless for as long as 75 min. The clinical application of multiparametric dynamic WB-PET is therefore limited due to the long scanning time, especially for oncology patients who cannot lie prostrate for long periods of time. Shorter scan times can, in addition to improving patient comfort, increase patient throughout, in turn increasing cost-effectiveness. To our knowledge, a scan duration optimization of multiparametric dynamic WB-PET has not yet been fully explored. The influence of quantitative performance in shortened dynamic PET was not investigated.

The first two silicon photomultiplier (SiPM)-based detectors with a time resolution of 214 ps time-of-flight (TOF) Biograph Vision PET/CT systems (Siemens Healthineers) in China were installed at Ruijing Hospital of Shanghai Jiaotong University and First Affiliated Hospital of Anhui Medical University, simultaneously. Furthermore, a multicenter retrospective study about Patlak parametric imaging was conducted at these two hospitals. Two different datasets were used to generate the multiparametric images: (i) 0–75 min dynamic PET series; (ii) 0–6 min + 60–75 min two short dynamic PET series. The 75-min continuous PET scan set (i) was noted as a standard acquisition protocol. The two-short-dynamic-scanning protocol contains two parts of data: the input function (0–6 min p.i.) and equilibrium activity (60–75 min p.i.). We evaluated the harmonization of Patlak parametric images generated from two short dynamic acquisitions and the standard scan protocol. The aim of this study was to explore the feasibility of shorter acquisition times for multiparametric imaging using the Biograph Vision PET/CT system.

## Methods

### Patient Population

Retrospective clinical studies about multiparametric dynamic WB-PET imaging were conducted in two hospitals in China from May 2020 to July 2021. Patients were scheduled for the dynamic WB scan protocol if they were deemed fit to lie still for 75 min while in the PET/CT scanner. Patients were required to fast for more than 6 h prior to scanning. Patients with a blood glucose level of greater than or equal to 198 mg/dl before ^18^F-FDG administration were excluded from participation in this study. All procedures performed in studies involving human participants were approved by the ethics committee of First Affiliated Hospital of Anhui Medical University and Ruijing Hospital of Shanghai Jiaotong University, and informed consent was obtained from all participants. Patient characteristics are outlined in **Table 1**.

**Table 1:** Clinical characteristics of the patient population and anatomical locations of the detected lesions.

Patient No.	Sex	Age (years)	Diagnosis	Detected Lesions
1	Male	71	Pancreatic adenocarcinoma	Pancreas (1), lymph node (1), bone (1)
2	Female	73	Lung carcinoma	Lungs (3), lymph nodes (4), bone (1)
3	Male	58	Hepatocellular carcinoma (postoperative)	Liver (1)
4	Male	62	Esophagus cancer (postoperative)	esophageal stoma (1), lymph nodes (5), pleura (3)
5	Male	72	Lung carcinoma	Lung (1), lymph nodes (3)
6	Female	36	Breast carcinoma (postoperative)	Chest wall (1), bones (2)
7	Male	49	Esophagus cancer	Esophagus (1), lymph nodes (5)
8	Male	64	Lung carcinoma	Lung (1), lymph nodes (5), thyroid (1)
9	Female	56	Pulmonary hamartoma	Lung (1), lymph nodes (4), colon (1)
10	Female	55	Pulmonary nodule	Lungs (2)
11	Male	68	Lung carcinoma	Lungs (3), lymph nodes (14), bone (2)
12	Female	71	Lung carcinoma	Lung (1), pleura (1)
13	Male	79	Lung carcinoma	Lungs (4), lymph nodes (5), paranephros (1)
14	Male	54	Lung carcinoma	Lung (1), lymph nodes (4)
15	Male	69	Lung carcinoma	Lung (1), lymph nodes (8), pleura (3)
16	Female	50	Ovarian cancer	Adnexa area (2), pelvic cavity (1), peritoneum (2), lung (1), lymph nodes (7)
17	Female	74	Lung carcinoma	Lung (1), lymph nodes (12), bone (1), thyroid (1)
18	Female	50	Lung carcinoma	Lung (1), lymph nodes (8),
19	Male	56	Pulmonary nodule	Lung (1)
20	Female	61	Pancreatic adenocarcinoma	Pancreas (1)
21	Male	81	Lymphoma	Pancreas (1), lymph node (3)

### Imaging Protocol

**Figure 1:**
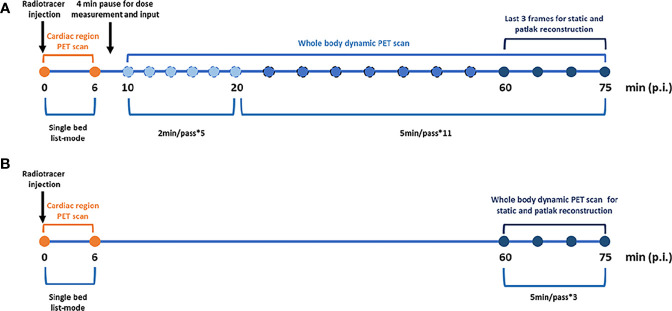
Imaging protocols. **(A)** Standard dynamic whole body Patlak parametric PET imaging (last 7 5min). **(B)** Two short dynamic whole body Patlak parametric PET imaging (0–6 min + 60–75 min post-injection).

Dynamic PET/CT scans were performed on a Siemens Biograph Vision scanner (Siemens Healthineers, Germany). First, a low-dose WB CT (an x-ray tube current of 43 mAs, a tube voltage of 100 kV, and a spiral pitch factor of 1) was performed from vertex to mid-thigh for acquisition correction. Then, PET data were acquired starting simultaneously with the injection of a weight-based dose of ^18^F-FDG (3.71 ± 1.05 MBq/kg). Patients underwent a scan protocol consisting of the following steps (**Figure 1**): (i) a 6-min dynamic single-bed list-mode PET acquisition centered over the cardiac region; (ii) a subsequent set of 16 WB-PET scans in continuous bed motion of 2 min/pass for the first 5 passes and 5 min/pass for the next 11 passes, in order to capture the late dynamics of the tracer in both the blood plasma and the tissues. The total PET scan time was about 75 min.

### Image Reconstruction

**Figure 2:**
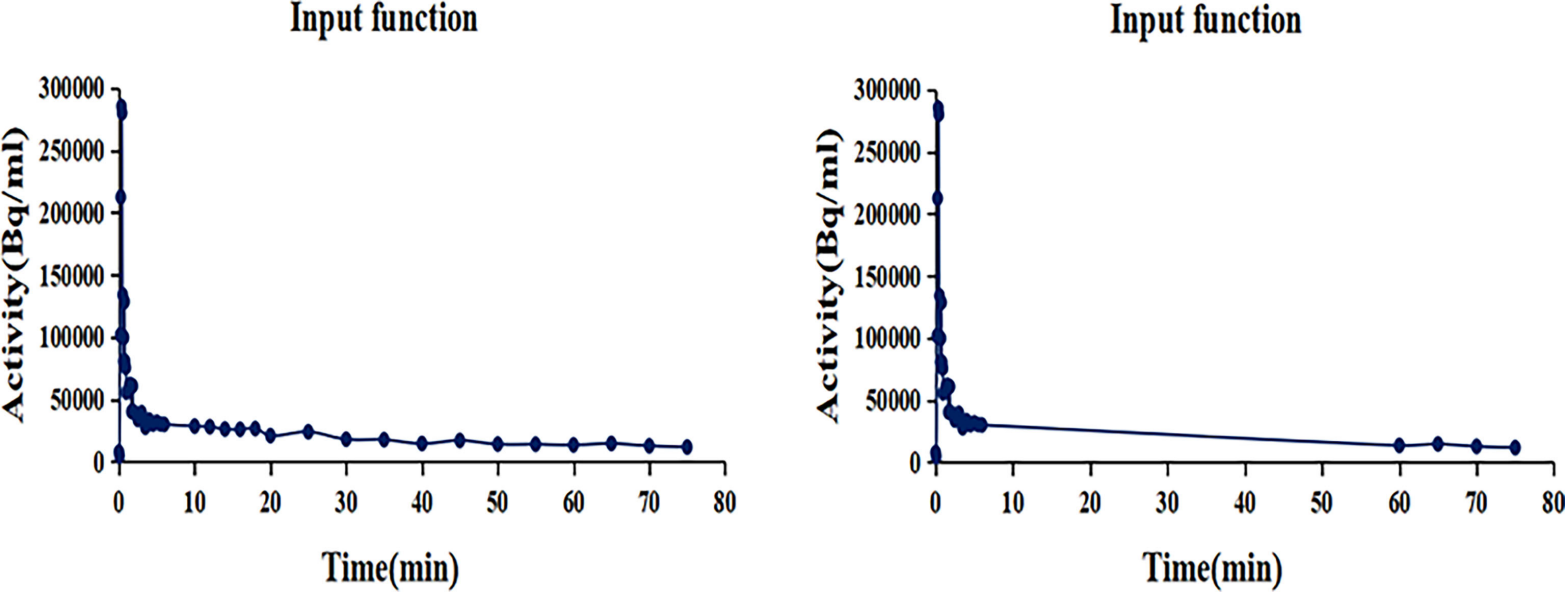
The input function TACs. (Left) IDIF was automatically generated from proximal descending aorta using dynamic PET series (0–75 min). (Right) TACs of proximal descending aorta were acquired from 0 to 6 min single bed list-mode PET images and the last three frames (60–75 min) dynamic PET series and were merged into one TAC in comma-separated values (CSV) files. The IDIF was then generated by importing these CSV files.

Parametric images of metabolic rate of FDG (MR_FDG_) and Patlak intercept (DV_FDG_) were generated using the nested directed Patlak reconstruction method on Siemens Biograph Vision workstation. After dynamic PET data acquisition, the automated multiparametric scan protocol automatically identified the descending aorta and placed a VOI from which an arterial image-derived input function (IDIF) was extracted from the selected dynamic PET series. The Patlak reconstruction (performs the Patlak transformation) to form the parametric images MR_FDG_ and DV_FDG_ used the last three frames of PET sinograms and used the IDIF acquired from the two datasets (**Figure 2**)—(i) the standard dataset (MR_FDG_-_std_ and DV_FDG_-_std_): IDIF was automatically generated from proximal descending aorta using dynamic PET series (0–75 min); (ii) the two-short-dynamic dataset (MR_FDG_-_tsd_ and DV_FDG_-_tsd_): TACs of proximal descending aorta were acquired from 0–6 min single bed list-mode PET images and the last three frames (60–75 min) of the dynamic PET series in TrueD (Siemens Healthineers) and were merged into one TAC in comma-separated values (CSV) files. The IDIF was then generated by importing these CSV files. Then, a straight line was fitted automatically by Siemens workstation using the Patlak method, and the slope of the fitted line was the Patlak *K*_i_ value applied to generate the Patlak *K*_i_ images. The patient’s blood glucose was obtained to calculate the metabolic rate of FDG (MR_FDG_), MR_FDG_ = *K*_i_ × blood glucose. Reconstruction parameters: Patlak recon method, OSEM True X + TOF, 4 iterations, 5 subsets, 220 matrices, with relative scatter correction and no Gaussian post filtering.

The conventional static clinical PET images were reconstructed using the last three dynamic PET frames (60 to 75 min) for SUV calculation (SUV_FDG_). Reconstruction parameters are as follows: OSEM True X + TOF, 4 iterations, 5 subsets, 220 matrices, with relative scatter correction and no Gaussian post filtering.

### Image Analysis

SUV_FDG_, MR_FDG_, and DV_FDG_ images were visually compared on workstation (syngo.via, Siemens Healthineers) by two nuclear medicine physicians. A volume of interest (VOI) was drawn over the target regions to obtain SUV_FDG_, MR_FDG_, and DV_FDG_ values. The VOIs of each lesion or normal organs on the standard MR_FDG_-_std_ and DV_FDG_-_std_ images were copied to the two short dynamic MR_FDG_-_tsd_ and DV_FDG_-_tsd_ images. Lesion VOIs were drawn using an automated delineation method with 41% of the maximum pixel value as segmentation threshold, while VOIs of normal organs were drawn using a sphere with a fixed diameter. Consistency of MR_FDG_ and DV_FDG_ from two different Patlak reconstructions was analyzed.

### Statistical Analysis

The statistical and graphical analysis of the extracted data were performed using MedCalc 20.03 (MedCalc Software, Ostend, Belgium). For inter-method correlation, Passing–Bablok regression was used including cusum test for linearity. Furthermore, inter-method agreement of MR_FDG_ and DV_FDG_ was analyzed using Bland–Altman plots. In all the tests, a *p*-value < 0.05 was considered statistically significant.

## Results

### Clinical and Multiparametric Image Characteristics

**Figure 3:**
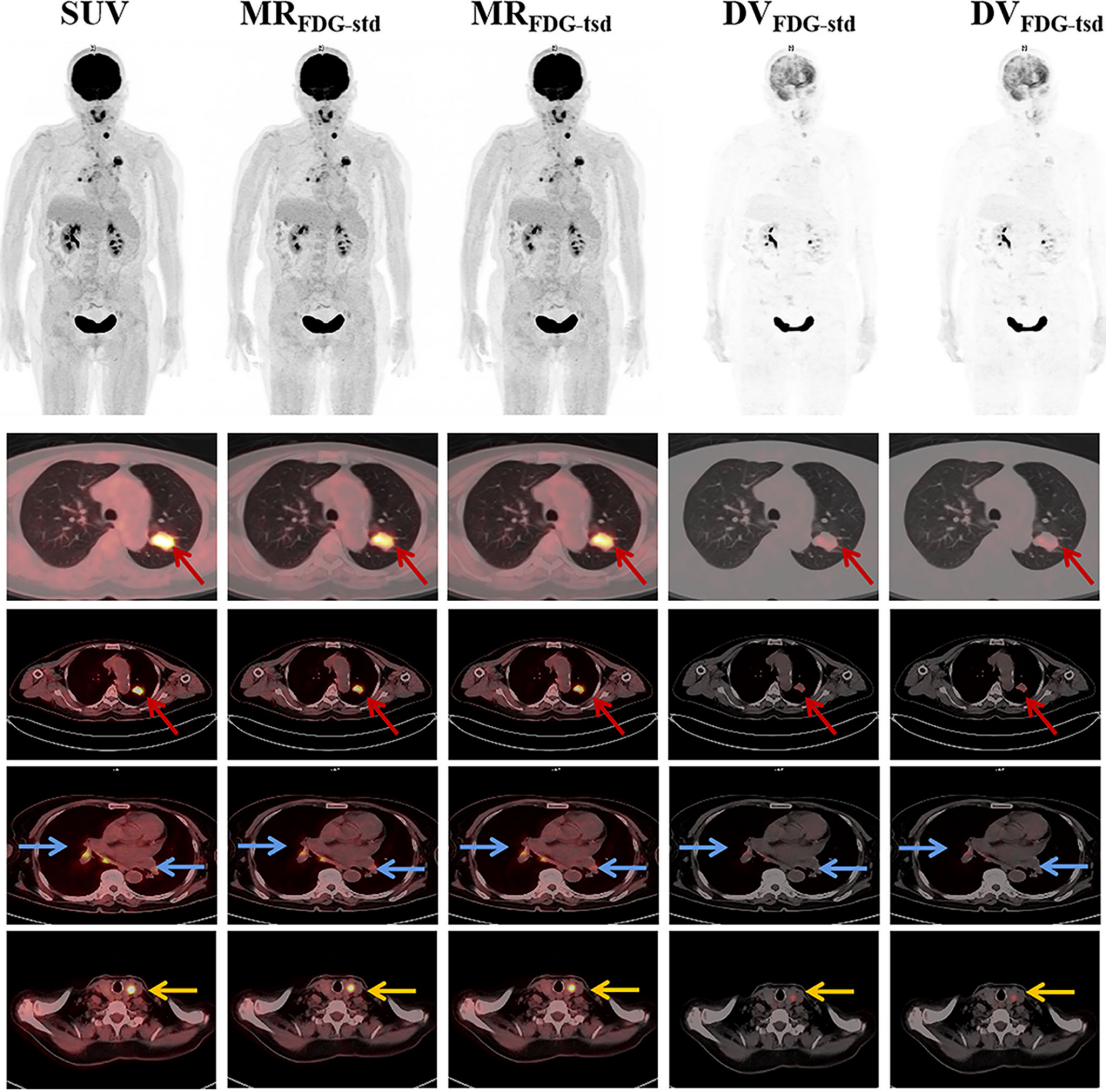
Representative static SUV_FDG_ images, and MR_FDG_ and DV_FDG_ images of standard and two short dynamic reconstruction groups. MR_FDG_ and DV_FDG_ images generated from both groups were found with good quality, and showed no visual distinction between the two reconstruction methods. The patient was diagnosed with pulmonary adenocarcinoma (red arrows). The PET scan revealed FDG uptake in multiple lymph nodes in the mediastinum and hilar(blue arrows). Incidental uptake of FDG in the thyroid gland was shown (yellow arrows).

**Figure 4:**
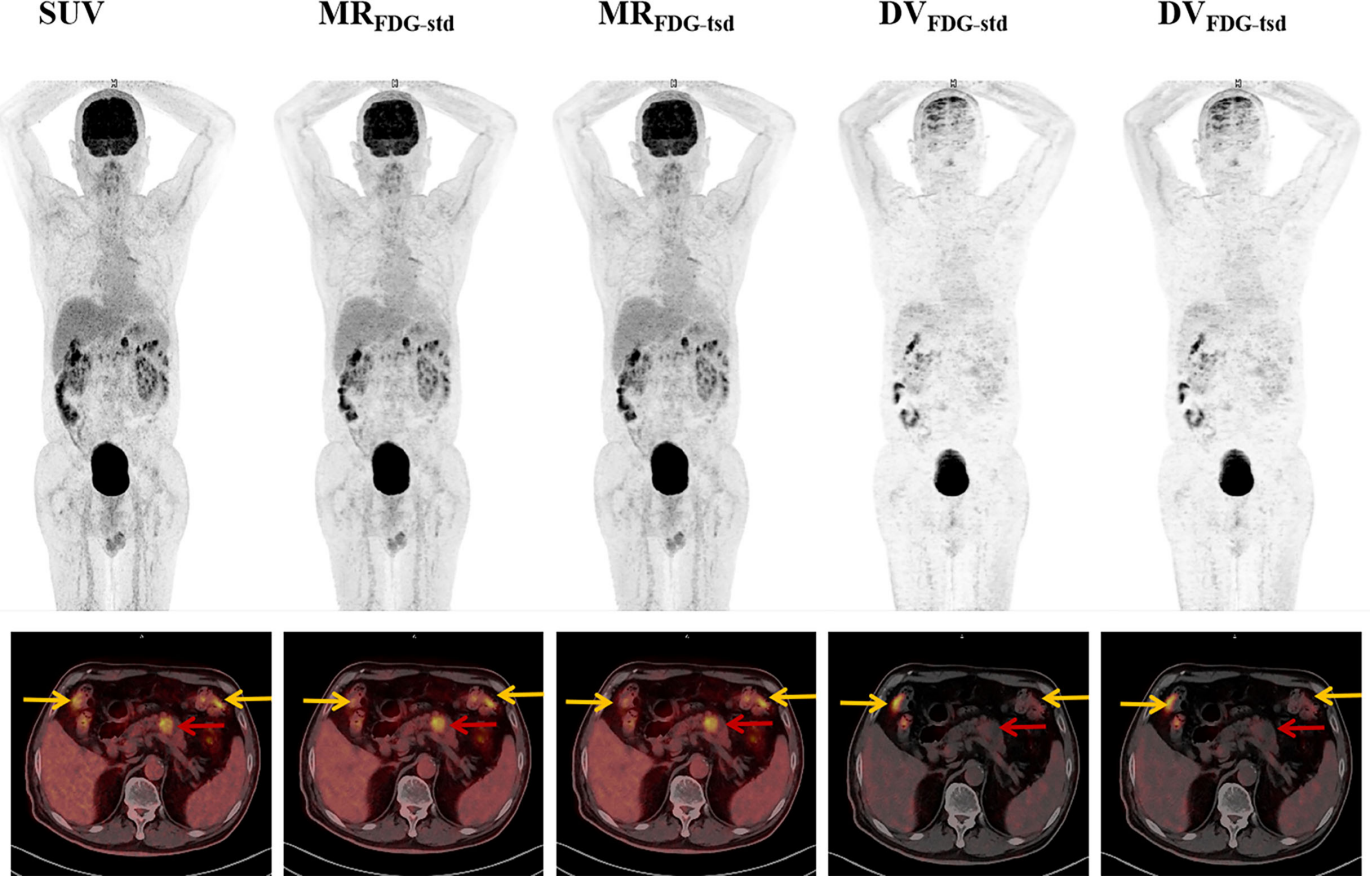
Representative MR_FDG_ and DV_FDG_ images of standard and two short dynamic reconstruction groups. The patient was diagnosed with pancreatic adenocarcinoma (red arrows). Intense focal tumoral uptake of FDG was shown on SUV and MR_FDG_ images, while there was no significant increased uptake on DV_FDG_ image. High physiological FDG uptake of colon was also found in types of images (yellow arrows).

Twenty-one patients were enrolled in this study (9 female and 12 male patients; mean age = 62.3 ± 11.4 years). **Table 1** summarizes the clinical indications and the anatomical locations of the lesions assessed for each patient. In this study, two different reconstruction methods were used to generate the multiparametric images of the standard MR_FDG_ and DV_FDG_ images (MR_FDG_-_std_ and DV_FDG_-_std_) and two short dynamic MR_FDG_ and DV_FDG_ images (MR_FDG_-_tsd_ and DV_FDG_-_tsd_). Both methods produced good-quality MR_FDG_ images and DV_FDG_ images, and there is no visual distinction of images between the two reconstruction methods (**Figures 3**, **4**). Compared with the static SUV_FDG_ images, the suppression of blood pool was found in several MR_FDG_ images for organs that have non-negligible fraction of blood pool compartment, such as the liver, spleen, large vessels, and renal pelvises. Due to the long scan time, the drawback of intestinal peristalsis artifacts in MR_FDG_ image and DV_FDG_ image that affected the diagnosis of intestinal lesions cannot be ignored.

### Comparison of MR_FDG_ and DV_FDG_ Values of Normal Organs in Different Reconstruction Groups

**Figure 5:**
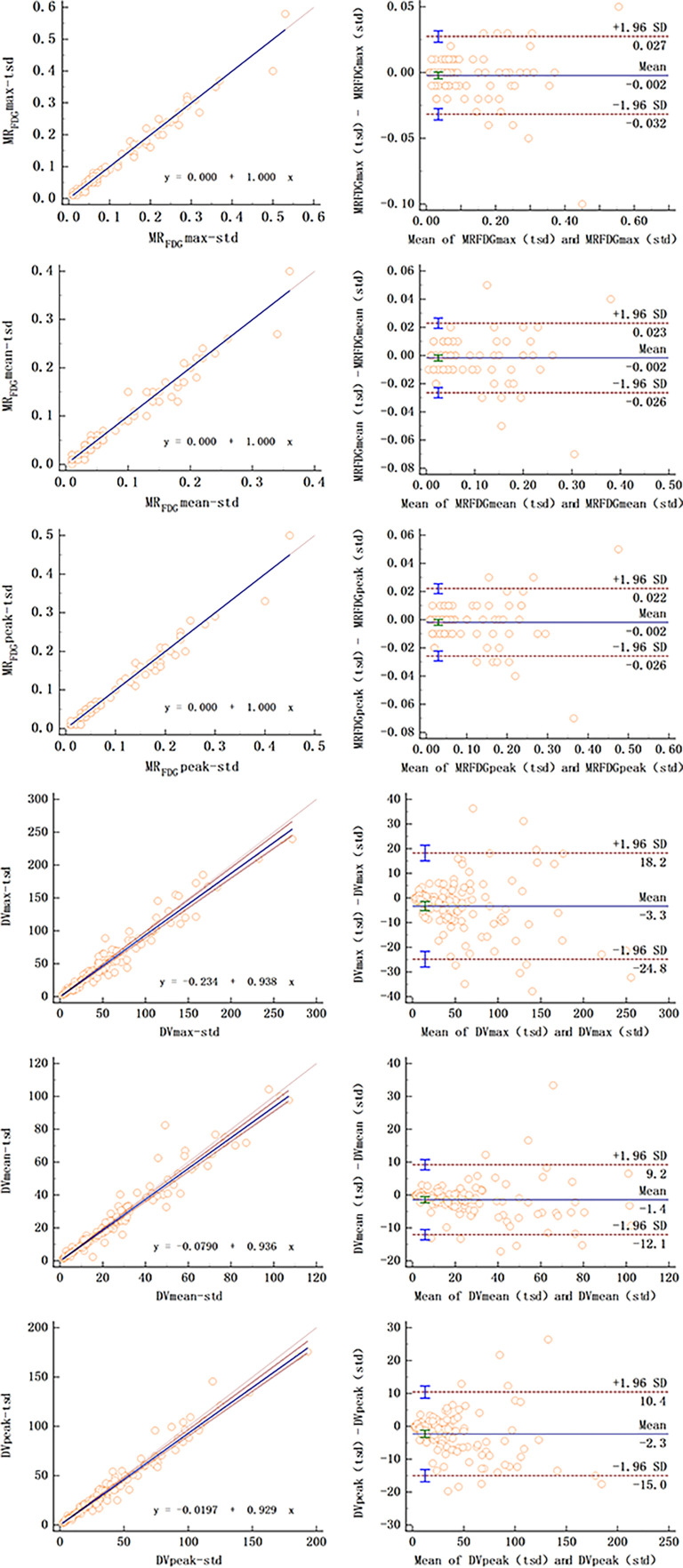
Passing–Bablok regression (left column) and Bland–Altman plot (right column) of MR_FDG_ and DV_FDG_ values of normal organs between standard and two short dynamic multiparametric images.

As summarized in **Table 2**, MR_FDG_ and DV_FDG_ showed a wide range of values among different organs with significant differences. The maximum, peak, or mean values of MR_FDG_ and DV_FDG_ in the brain and heart wall were relatively high, while those of the lung were the lowest. When comparing MR_FDG_ and DV_FDG_ values of normal organs between the standard group and the two-short-dynamic group, no significant difference was observed. The null hypothesis assuming non-linearity was rejected in MR_FDG_ and DV_FDG_ values. There is almost no difference between MR_FDG_-_tsd_ and MR_FDG-std_; the intercept A was 0 and the Slope B was 1. There is a slight variation between DV_FDG-tsd_ and DV_FDG-std_. The value 0 was included in the confidence interval of intercept A in DV_FDG_ values; however, the value 1 in the confidence interval of Slope B was not included. The Bland–Altman plot showed that the mean bias of MR_FDG-_max, MR_FDG-_mean, and MR_FDG-_peak was -0.002 (95% CI: -0.032–0.027), -0.002 (95% CI: -0.026–0.023), and -0.002 (95% CI: -0.026–0.022), respectively. The mean bias of DV_FDG-_max, DV_FDG-_mean, and DV_FDG-_peak was -3.3 (95% CI: -24.8–18.2), -1.4 (95% CI: -12.1–9.2), and -2.3 (95% CI: -15–10.4), respectively (**Table 3** and **Figure 5**).

**Table 2:** MR_FDG_, DV_FDG_, and SUV values of normal organs.

		Brain	Lung	Liver	Spleen	Heart wall	Bone	Muscle
MR_FDG-std_	Max	0.23 ± 0.06	0.01 ± 0.01	0.06 ± 0.02	0.06 ± 0.12	0.19 ± 0.16	0.06 ± 0.02	0.02 ± 0.01
	Mean	0.17 ± 0.05	0.01 ± 0.00	0.04 ± 0.01	0.04 ± 0.01	0.12 ± 0.11	0.04 ± 0.02	0.01 ± 0.01
	Peak	0.19 ± 0.05	0.01 ± 0.00	0.05 ± 0.01	0.05 ± 0.01	0.15 ± 0.13	0.05 ± 0.020	0.02 ± 0.01
MR_FDG-tsd_	Max	0.22 ± 0.07	0.01 ± 0.01	0.06 ± 0.02	0.06 ± 0.01	0.17 ± 0.15	0.06 ± 0.02	0.02 ± 0.01
	Mean	0.16 ± 0.55	0.01 ± 0.00	0.04 ± 0.01	0.04 ± 0.01	0.12 ± 0.11	0.04 ± 0.02	0.01 ± 0.00
	Peak	0.19 ± 0.06	0.01 ± 0.00	0.05 ± 0.02	0.04 ± 0.01	0.14 ± 0.12	0.05 ± 0.02	0.02 ± 0.01
DV_FDG-std_	Max	136.42 ± 60.33	11.22 ± 6.22	58.92 ± 14.13	45.32 ± 10.92	95.64 ± 49.36	36.52 ± 14.48	17.41 ± 8.00
	Mean	59.17 ± 21.62	4.14 ± 2.18	28.35 ± 6.95	21.89 ± 3.97	48.83 ± 27.61	14.62 ± 6.38	6.96 ± 3.21
	Peak	99.19 ± 40.14	7.23 ± 4.16	39.45 ± 8.57	31.15 ± 6.73	62.80 ± 31.15	22.30 ± 9.13	10.69 ± 4.00
DV_FDG-tsd_	Max	126.50 ± 59.01	10.80 ± 5.61	56.84 ± 19.28	41.65 ± 13.19	87.35 ± 49.36	34.42 ± 14.26	16.25 ± 9.30
	Mean	55.78 ± 24.62	4.02 ± 2.01	26.88 ± 8.34	19.75 ± 6.49	45.34 ± 25.56	13.60 ± 6.11	6.45 ± 3.24
	Peak	92.11 ± 41.65	6.83 ± 3.53	37.49 ± 11.53	28.93 ± 7.70	56.83 ± 28.99	20.71 ± 8.39	10.03 ± 4.72
SUV	Max	9.62 ± 2.22	0.63 ± 0.32	2.92 ± 0.57	2.54 ± 0.46	7.63 ± 5.60	2.65 ± 1.00	0.92 ± 0.22
	Mean	7.35 ± 1.87	0.40 ± 0.18	2.23 ± 0.39	2.01 ± 0.31	5.47 ± 4.04	1.93 ± 0.86	0.63 ± 0.14
	Peak	8.58 ± 1.89	0.54 ± 0.26	2.49 ± 0.45	2.18 ± 0.33	6.29 ± 4.59	2.24 ± 0.87	0.76 ± 0.16

MRFDG: umol/min/ml, DVFDG: %.

**Table 3:** Passing–Bablok regression of MR_FDG_ and DV_FDG_ of normal organs between different reconstruction groups.

	Intercept A (CI)	Slope B (CI)	Random differences (CI)	Cusum *p*-value	Spearman rho (CI); *p*-value
MR_FDG-_max	0.000 (0.000–0.000)	1.000 (1.000–1.000)	0.011 (-0.021–0.021)	*p* = 0.78	0.980 (0.972–0.986); *p* < 0.0001
MR_FDG-_mean	0.000 (0.000–0.000)	1.000 (1.000–1.000)	0.009 (-0.018–0.018)	*p* = 0.97	0.968 (0.955–0.977); *p* < 0.0001
MR_FDG-_peak	0.000 (0.000–0.000)	1.000 (1.000–1.000)	0.009 (-0.017–0.017)	*p* = 0.96	0.977 (0.968–0.983); *p* <0.0001
DV_FDG-_max	-0.234 (-1.117–0.620)	0.938 (0.906–0.978)	7.631 (-14.958–14.958)	*p* = 0.86	0.973 (0.962–0.980); *p* < 0.0001
DV_FDG-_mean	-0.079 (-0.464–0.157)	0.936 (0.912–0.967)	3.909 (-7.661–7.661)	*p* = 0.24	0.979 (0.971–0.985); *p* < 0.0001
DV_FDG-_peak	-0.020 (-0.494–0.431)	0.929 (0.906–0.962)	4.591 (-8.998–8.998)	*p* = 0.86	0.980 (0.973–0.986); *p* < 0.0001

### Comparison of MR_FDG_ and DV_FDG_ Values of Lesions in Different Reconstruction Groups

**Figure 6:**
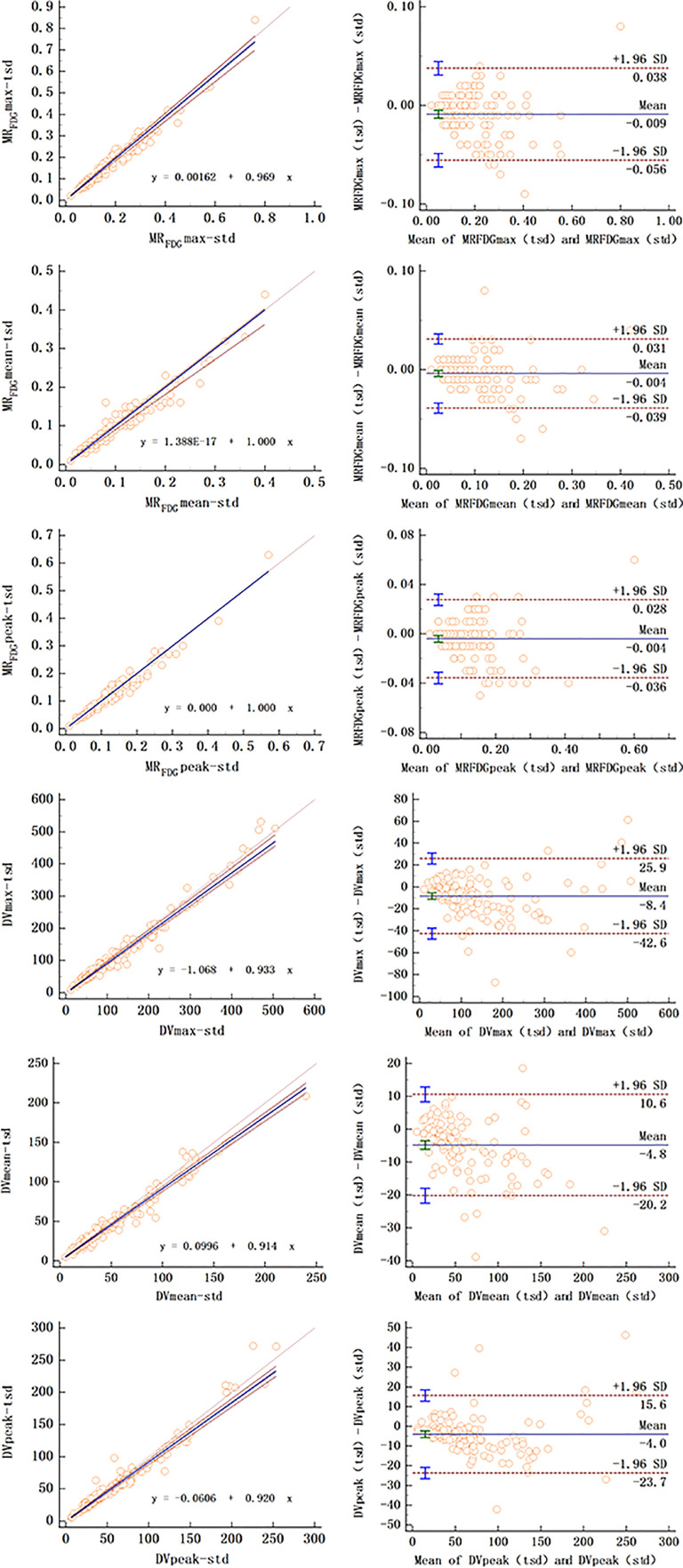
Passing–Bablok regression (left column) and Bland–Altman plot (right column) of MR_FDG_ and DV_FDG_ values of lesions between standard and two short dynamic multiparametric images.

For MR_FDG_ and DV_FDG_ values of lesions, there was substantial correlation between two reconstruction groups. Passing–Bablok regression and Bland–Altman plots demonstrate the inter-method agreement and show that differences of MR_FDG_ and DV_FDG_ were acceptably small in the cohort (**Table 4** and **Figure 6**).

**Table 4:** Passing–Bablok regression of MR_FDG_ and DV_FDG_ of lesions between different reconstruction groups.

	Intercept A (CI)	Slope B (CI)	Random differences (CI)	Cusum *p*-value	Spearman rho (CI); *p*-value
MR_FDG-_max	0.002 (0.000–0.006)	0.969 (0.920–1.000)	0.015 (-0.030–0.030)	*p* = 0.21	0.982 (0.975–0.987); *p* < 0.0001
MR_FDG-_mean	1.388E-17 (0.000–0.005)	1.000 (0.906–1.000)	0.013 (-0.025–0.025)	*p* = 0.30	0.962 (0.947–0.973); *p* < 0.0001
MR_FDG-_peak	0.000 (0.000–0.000)	1.000 (1.000–1.000)	0.012 (-0.023–0.023)	*p* = 0.66	0.977 (0.968–0.984); *p* < 0.0001
DV_FDG-_max	-1.068 (-3.407–1.190)	0.933 (0.909–0.970)	13.478 (-26.417–26.417)	*p* = 0.73	0.982 (0.974–0.987); *p* < 0.0001
DV_FDG-_mean	0.100 (-0.825–0.962)	0.914 (0.889–0.935)	5.027 (-9.852–9.852)	*p* = 0.45	0.976 (0.966–0.983); *p* < 0.0001
DV_FDG-_peak	-0.061 (-1.452–1.072)	0.920 (0.893–0.946)	7.849 (-15.384–15.384)	*p* = 0.86	0.977 (0.968–0.984); *p* < 0.0001

The null hypothesis assuming non-linearity was rejected in all cases. The Bland–Altman plot showed that the mean bias of MR_FDG-_max, MR_FDG-_mean, and MR_FDG-_peak was -0.009 (95% CI: -0.056–0.038), -0.004 (95% CI: -0.039–0.031), and -0.004 (95% CI: -0.036–0.028), respectively. The mean bias of DV_FDG-_max, DV_FDG-_mean, and DV_FDG-_peak was -8.4 (95% CI: -42.6–25.9), -4.8 (95% CI: -20.2–10.6), and -4.0 (95% CI: -23.7–15.6), respectively.

## Discussion

Dynamic PET enables full quantitative imaging (as opposed to SUV, which is semi-quantitative) by producing additional multiparametric images: the MR_FDG_ image, which is the rate of irreversible uptake, and the Patlak intercept (DV) image, which is the apparent distribution volume of the non-metabolized FDG (24). The clinical application of multiparametric dynamic WB-PET is seriously limited due to the long scanning time. Our study evaluated the harmonization of Patlak parametric images reconstructed using two short dynamic subsets including the first 0–6 min and 60–75 min scans post injection compared to the standard parametric images derived from the complete 0–75 min dynamic scans. The feasibility of shorter acquisition times on multiparameter collection was investigated.

The results showed that both reconstruction groups produced MR_FDG_ images and DV_FDG_ images with good quality, and without visual distinction of images between the two reconstruction methods. Compared with the static SUV_FDG_ images, the suppression of blood pool is obvious in MR_FDG_ images for organs that have non-negligible fraction of blood pool compartment, such as the liver, spleen, large vessels, and renal pelvises. This feature of MR_FDG_ images commonly results in higher contrast for lesions located close to these anatomical structures as reported in previous studies (21, 25). Due to the long scanning time, the only drawback of MR_FDG_ image and DV_FDG_ image was the deviation caused by intestinal peristalsis artifacts, which affected the diagnosis of intestinal lesions, which is consistent with the previous research (26). The visual performance of normal organs and lesions was similar in MR_FDG_ images and DV_FDG_ images of two reconstruction groups. For quantification evaluation, the overall maximum, mean, and peak values of MR_FDG_ and DV_FDG_ of normal organs and lesions between two reconstruction groups showed significant linear correlation, while no significant differences were found in Passing–Bablok regression analysis. The uniformity of MR_FDG_, DV_FDG_, and SUV_FDG_ values between the group with two short dynamic acquisitions and the group with standard 0–75 min continuous dynamic scans was visualized using Bland–Altman plots. This indicates that our proposed method might have potential to improve clinical workflow for WB Patlak parametric imaging.

Several attempts have been made to reduce duration of dynamic PET scans. Yao et al. (27) proposed a simplified WB scanning protocol that utilizes both population-based input function and model-based input function for WB Patlak image reconstruction. Feng et al. (28) demonstrated that with the use of an ultrahigh-sensitivity total-body PET scanner, it is possible to achieve WB parametric image reconstruction using only the early stage of the scan (within the first 2 min post injection). Wu et al. (29) explored and proved the possibility of shortened acquisition time for parametric imaging of *K*_i_ in FDG PET scan employing the nonlinear estimation approach with 7 patients scanned on a total-body PET scanner. Wu et al. (30) generated parametric *K*_i_ images of FDG PET using two 5-min scans, which showed higher quantification accuracy with respect to standard *K*_i_ than relative SUV change. In any case, the influence of quantitative performance in shortened dynamic PET was not investigated. In this study, the two short dynamic scans were obtained from complete 75-min dynamic sets with the patient remaining in the same position in the scanner. The dataset between the early and late scans was cut off. The obliteration of data during the intermediate period was a big perturbation for IDIF, which could influence the quantitative parameters. The aim of the present study was to investigate whether two short dynamic scans can achieve reliable multiparametric images without sacrificing quantitative performance.

One of the limitations of two-short-dynamic scanning is the patient motion artifact. Tomohito Kaji et al. (31) suggested to eliminate the frames with motion, which seems to be particularly useful for motion artifact. In this study, the two short dynamic scans were obtained from complete 75-min dynamic sets with the patient remaining in the same position in the scanner. In clinical two-short-dynamic imaging, the patient would leave the scanner between the early and late scans, which can increase patient throughout. This means that two aorta VOIs will need to be used to generate the full IDIF from the two scans, and two CT scans would be required for accurate attenuation correction of the two PET images, with the first scan only requiring an axial FOV that covers the cardiac region (e.g., single bed acquisition). Input VOIs can be acquired using the reconstruct images of the early and late scans drawn over a large arterial vessel, like the descending aorta. The second CT covering the whole body (from vertex to mid-thigh) will be performed before the static PET scanning in order to avoid misalignment. Note that the WB dynamic CBM scan is only needed for the second PET/CT scan to generate the tissue TACs.

## Conclusion

This study shows that two short dynamic scans for multiparametric PET image collection enable a reduction of scan time duration without sacrificing quantitative performance. The maximum, mean, and peak values of MR_FDG_ and DV_FDG_ reconstructed using standard and two-short-dynamic-scanning data are statistically consistent.

## Data Availability Statement

The original contributions presented in the study are included in the article/supplementary material. Further inquiries can be directed to the corresponding authors.

## Ethics Statement

All procedures performed in studies involving human participants were approved by the ethics committee of First Affiliated Hospital of Anhui Medical University and Ruijing Hospital of Shanghai Jiaotong University, and informed consent was obtained from all participants. The patients/participants provided their written informed consent to participate in this study. Written informed consent was obtained from the individual(s) for the publication of any potentially identifiable images or data included in this article.

## Author Contributions

HW and HX conceived the idea of the study. HW, YM, and WY collected the data. HW, YM, GZ, TW, GY, and XZ performed image analysis. HW wrote the manuscript and performed the statistical analysis. HX and BL edited the manuscript. All authors contributed to the article and approved the submitted version.

## Funding

The study was funded by the National Natural Science Foundation of China (Nos. 81801736 and 81971643).

## Conflict of Interest

The authors declare that the research was conducted in the absence of any commercial or financial relationships that could be construed as a potential conflict of interest.

## Publisher’s Note

All claims expressed in this article are solely those of the authors and do not necessarily represent those of their affiliated organizations, or those of the publisher, the editors and the reviewers. Any product that may be evaluated in this article, or claim that may be made by its manufacturer, is not guaranteed or endorsed by the publisher.
